# Exploring Dietary- and Disease-Related Influences on Flatulence and Fecal Odor Perception in Inflammatory Bowel Disease

**DOI:** 10.3390/jcm14010137

**Published:** 2024-12-29

**Authors:** Lea Pueschel, Sonja Nothacker, Leonie Kuhn, Heiner Wedemeyer, Henrike Lenzen, Miriam Wiestler

**Affiliations:** 1 Department of Gastroenterology, Hepatology, Infectious Diseases and Endocrinology, Hannover Medical School, 30625 Hannover, Germany; pueschel.lea@mh-hannover.de (L.P.); kuhn.leonie@mh-hannover.de (L.K.); wedemeyer.heiner@mh-hannover.de (H.W.); or h.lenzen@skbs.de (H.L.); 2 School for Dietitians, Hannover Medical School, 30625 Hannover, Germany; nothacker.sonja@mh-hannover.de; 3 Department of Gastroenterology, Hepatology, Interventional Endoscopy and Diabetology, Academic Teaching Hospital Braunschweig, 38126 Braunschweig, Germany

**Keywords:** inflammatory bowel disease, malodor perception, flatulence, highly processed foods, healthy control

## Abstract

**Background/Objectives**: Inflammatory bowel disease (IBD) affects gastrointestinal function and may alter fecal and flatulence odor (intestinal odor) due to changes in inflammation, the gut microbiome, and metabolism. Investigating the relationship between dietary habits and intestinal odor in IBD is critical given the relationship between diet, gut health, and microbiome diversity. **Methods**: We performed a cohort analysis of a monocentric, cross-sectional study at a tertiary referral center and compared the perception of fecal and flatulence odor in 233 IBD patients (n = 117 women) with that of 96 healthy controls (HCs) (n = 67 women). In addition to a short screening questionnaire on highly processed foods (sQ-HPF), dietary behavior (Food Frequency Questionnaire (FFQ)), clinical (HBI, PMS) and biochemical (CRP, fecal calprotectin) parameters of disease activity, and adherence to a Mediterranean diet were assessed. **Results**: A notable predisposition towards elevated levels of intestinal malodor was identified in the IBD cohort when compared to the HC group. The analysis of dietary behavior in conjunction with intestinal malodor revealed more pronounced associations in the HC collective than in the IBD collective. The data further indicated that, in comparison to those in remission, IBD individuals with an active disease status exhibited a higher prevalence of intestinal malodor. In an adjusted logistic regression analysis of the influence of disease- and diet-specific factors on flatulence and fecal malodor in IBD, male sex was identified as a significant risk factor. **Conclusions**: This study highlights the significance of dietary factors in the management of IBD symptoms, with a particular focus on flatulence and fecal odor. Individuals with IBD demonstrated a higher propensity for intestinal malodor compared to HC, with active disease status further amplifying this prevalence. Dietary behavior showed stronger associations with malodor in the HC group than in IBD individuals, suggesting distinct interaction patterns between diet and gut health in these populations.

## 1. Introduction

In the gastrointestinal tract, numerous gases are produced through chemical reactions or the metabolic processes of resident microbiota. These include hydrogen (H_2_), methane (CH_4_), and carbon dioxide (CO_2_), along with smaller quantities of trace gases such as hydrogen sulfide (H_2_S), nitric oxide (NO), and sulfur-based compounds [1]. The primary source of these gases is bacterial fermentation of dietary components. Despite ongoing research into the impact of dietary substrates on the human body, our knowledge of the dynamic processes involved in how food is metabolized in the gut remains limited and rudimentary. In the general population, the type and strength of flatulence and fecal odor are known to be influenced by nutritional choices—a diet with a high fiber or protein content can increase or change the odor that is perceived [1,2]. In addition, food intolerances and fermentable oligosaccharides, as contained in wheat or onions, can cause increased intestinal odor [3]. However, a change in odor can also be health-related: changes in fecal volatile organic compound (VOC) have been suggested as an indicator of inflammatory bowel diseases (IBDs) [4,5] as well as changes in disease progression for IBD [6]. It is well documented that IBD, including Crohn’s disease (CD) and ulcerative colitis (UC), has a significant impact on the function of the digestive tract, resulting in a range of symptoms, including alterations in the odor of feces and flatulence [7]. The underlying pathophysiological changes associated with IBD, such as inflammation, malabsorption, and alterations in the gut microbiota, are thought to be primarily responsible for these distinct changes [7]. The presence of specific compounds, including hydrogen sulphide, which is produced during the fermentation of undigested food in the colon, is often linked to IBD [8]. Inflammation, which interferes with normal digestion, further exacerbates this process in individuals with IBD. An increased risk of developing inflammation and of experiencing an exacerbation of inflammation, meanwhile, has been associated with ultra-processed foods in general [9,10], but there may also be an increased risk of IBD from ultra-processed foods and dietary emulsifiers such as carboxymethylcellulose [11]. Achieving a sustained remission remains one of the most important treatment goals in IBD to prevent disease progression and subsequent complications. Ileocolonoscopy is currently regarded as the gold standard for assessing mucosal inflammation in IBD substantiated by histological assessment. However, frequent colonoscopies pose a significant burden for patients and are costly and resource-intensive for healthcare systems. As a result, there is a clear need for non-invasive markers that can reliably predict changes in disease status at an early stage. Accurately predicting changes in disease state at an early stage enables timely adjustments to treatment, which in turn enhances disease outcomes and helps to minimize drug-related side effects. Especially patient-reported outcome measures (PROMS) can be a fast point of care tool to assess patient characteristics on time. In this context, especially fecal and flatulence odor perception might have advantages, as it is non-invasive, frequent, and easy to apply. As only limited data exist on intestinal odor perception and the association with dietary habits and IBD activity, the objective of this study was to gain further insight into these specific relationships in individuals with IBD.

## 2. Materials and Methods

The study design is in accordance with the ethical standards set forth in the Declaration of Helsinki (2013). This monocentric study was conducted at a tertiary referral center and has been approved by the Ethics Committee of the Hannover Medical School (10847_BO_S_2023) and is registered in the German Clinical Trials Register (DRKS) under DRKS00032771 (https://drks.de/search/en/trial/DRKS00032771 (accessed on 26 November 2024)).

### 2.1. Participants and Setting

Between October 2023 and October 2024, a total of 275 IBD patients were screened for study participation at Hannover Medical School. Written informed consent was required before inclusion in the study. Eligibility criteria were a confirmed diagnosis of either UC or CD and a disease duration of at least three months. Individuals with conditions that precluded an assessment of the nature, extent, and potential consequences of the study were excluded. Patients younger than 18 were not eligible for study participation.

### 2.2. Healthy Controls

A total of 101 individuals were selected to participate in this monocentric, cross-sectional study between October 2023 and October 2024. Written informed consent was required before inclusion in the study. Diagnosis of inflammatory bowel disease was an exclusion criterion for enrollment in the healthy control cohort. Individuals with conditions that precluded an assessment of the nature, extent, and potential consequences of the study were excluded. Individuals younger than 18 were not eligible for study participation.

### 2.3. Variables and Definitions

#### 2.3.1. Data Sources/Measurements

Data were collected via an online survey that was only accessible for study participants who provided written consent. Types of questions included information on sex and gender identity, body type (weight, height), age, marital status, employment status, and more. All individuals were asked to complete a food frequency questionnaire (FFQ) [12] and the German version of the Screening Questionnaire of Highly Processed Food Consumption (sQ-HPF) [13]. IBD-specific history, therapies, surgical history, and comorbidities were also included in the online questionnaire. Disease activity was assessed in investigator-administered interviews using either the German version of the Harvey–Bradshaw Index (HBI) [14] for CD patients or the German version of the Mayo Score, PMS [15], for UC patients. Disease extent was determined using the Montreal classification for CD patients and the anatomical pattern for UC patients [16].

#### 2.3.2. Advanced Therapies

Current treatment with advanced therapies (ADT), including TNF-, interleukin (IL) 12/23 and interleukin (IL) 23, integrin antagonists, JAK inhibitors, and Sphingosine-1-phosphate receptor modulators, was documented for each patient and coded as a binary variable (current ADT treatment: yes/no) for logistic regression analysis.

#### 2.3.3. Food Frequency Questionnaire Variables and Macronutrients

For Food Frequency Questionnaire (FFQ) scoring, average daily amounts of individual foods and beverages were calculated [12], while nutrient intakes were calculated using Federal Food Code (BLS) reference data [17]. Estimated energy intake (EEI) is reported in kilojoule (kJ), as is the sex-specific resting energy expenditure (REE).

#### 2.3.4. Mediterranean Diet Score

Mediterranean diet adherence was adjusted from Trichopoulou et al. [18] based on sex means for selected food groups. One point was awarded for each positively associated food (vegetables, pulses and legumes, fruit and nuts, cereals, fish) if the consumption was equal to or higher than the mean value. For each of the negatively associated foods (meat, poultry, dairy products), one point was awarded if the consumption was less than the mean value. Fat intake was calculated based on the FFQ dietary analysis using the ratio of monounsaturated fat (g) to saturated fat (g). For ethanol intake, Trichopoulou et al.’s [18] sex-specific values were used. The total MDS score is between 0 and 9, with 9 representing maximum compliance with the Mediterranean diet.

#### 2.3.5. Malnutrition Universal Screening Tool

The German version of the Malnutrition Universal Screening Tool (MUST) has been used to identify adults who are potentially at risk of malnutrition [19,20]. The scoring is based on body mass index (BMI), unplanned loss of weight within the last 3 to 6 months, and acute illness with an involuntary fasting period of at least 5 days. The total score ranges from 0 to 6, with 0 indicating low risk and anything above 2 indicating high individual risk of malnutrition.

#### 2.3.6. Disease Activity

Entity-specific Disease Activity Index cut-offs were used to determine disease activity and remission. For the binary assessment of disease activity, remission was defined as a Harvey–Bradshaw Index (HBI) of <5 [14] in CD patients or a partial Mayo score (PMS) of 0–1 [15] in UC patients.

#### 2.3.7. Screening Questionnaire of Highly Processed Food Consumption

The percentage of habitual, highly processed food consumption out of total intake in grams per day was estimated using the German version of the Screening Questionnaire of Highly Processed Food Consumption (sQ-HPF) [13]. [Manuscript for the translated and validated German version of the sQ-HPF currently under review].

#### 2.3.8. Highly Processed and Ultra-Processed Foods

All FFQ items corresponding to the translated version of the sQ-HPF were selected to calculate intake of highly processed foods and beverages. In comparison, with the sole exception of canned/preserved fruits, the intake of ultra-processed foods and drinks (UPFD) was calculated by critically identifying the class 4 NOVA food classification [21] items of the FFQ. For both the HPF and the UPFD, the daily energy content (kJ/d) and the total daily weight (g/d) were calculated.

#### 2.3.9. Gastrointestinal Surgery

Surgery status was recorded and used as a binary variable (yes/no) in the analysis as a potential confounding and adjustment factor. Additionally, a binary variable for pouch/stoma (yes/no) was created.

#### 2.3.10. Flatulence and Fecal Odor Perception

All study participants were asked whether they perceive individual flatulence and fecal odor as exceptionally strong (malodorous), with the answers being coded as binary variables (yes/no). Flatulence and fecal malodor were used as outcome variables for logistic regression analysis.

#### 2.3.11. Laboratory Values

As part of the screening visit, biomaterials (blood and stool samples) were collected during routine outpatient clinic visits. Laboratory values included C-reactive protein (CRP) (mg/L) and calprotectin (mg/kg).

### 2.4. Statistical Analysis

The statistical analysis was conducted using the SPSS Statistics software, version 29.0.1.0 (SPSS, IBM, Armonk, NY, USA), and GraphPad PRISM, version 10.4.0 (GraphPad Software, Boston, MA, USA). The Shapiro–Wilk test was used to assess normal distribution. Categorical outcomes are reported as totals and proportions. For group comparisons of categorical variables, the Bonferroni correction was applied to Fisher’s exact test. Unless otherwise stated, all statistical tests were performed two-sided. Significance levels are indicated in figures as one asterisk for *p* = 0.05, two for *p* = 0.01, and three for *p* < 0.001. Student’s *t*-test was used for odor-related comparisons of dietary variables within the healthy and IBD groups. Student’s *t*-test was also used for odor-related comparisons of dietary variables between the groups (healthy vs. IBD). For IBD only, data were further analyzed using binary logistic regression to assess the probability of an association between disease and diet-related events with (a) flatulence malodor and (b) fecal malodor. The odds ratio (OR), 95% confidence interval (CI), and level of significance (p) are reported. To ensure the reliability of the adjusted model, we tested for multicollinearity of the variables associated with each other in the binary logistic regression model. Consequently, the variables GI surgery and Pouch/Stoma were not used simultaneously in the adjusted model. Results of logistic regression analysis (univariate and adjusted (multivariate)) are available as supplementary data (Tables S1 and S2: Adjusted logistic regression analysis of the influence of disease-specific and dietary-specific factors […]). Meanwhile, the results of the fully adjusted multivariate logistic regression are reported on in the results section. Goodness of fit for the fully adjusted logistic regression model with the outcome flatulence malodor was assessed via Omnibus Tests of Model Coefficients (*p* < 0.001), R2 (Nagelkerkes: 0.485; Cox & Snell: 0.363), and the Hosmer–Lemeshow test (*p* = 0.459). The model performance was assessed via the classification table, which showed an overall percentage of 81.4%. Goodness of fit for the fully adjusted logistic regression model with the outcome fecal malodor was assessed via Omnibus Tests of Model Coefficients (*p* < 0.001), R2 (Nagelkerkes: 0.475; Cox & Snell: 0.350), and the Hosmer–Lemeshow test (*p* = 0.776). The model performance was assessed via the classification table, which showed an overall percentage of 80.5%.

#### 2.4.1. Confounders and Bias

Each regression model was adjusted for confounders, including disease entity, sex, remission status, pouch/stoma, vegetarian status, and age, to control for potential confounding variables. A detailed description of the adjustment factors can be found in the Supplementary Materials. [Table S1A,B: Adjustment factors for Outcome […]] Prior to data analysis, cases were screened for individuals currently nursing, revealing n = 2 IBD individuals and n = 2 HC individuals who were actively nursing during study participation. Dietary intake was immensely higher for nursing individuals, to avoid distortion of further analysis, data from all nursing individuals were consequently excluded. The estimated effect size (g) is reported in addition to the statistical significance (p), as (g) is independent of sample size, to account for the small subgroup study population of healthy controls. Recall surveys are susceptible to bias, and misreporting of dietary intake in patient-reported outcomes is not uncommon [22]. Over-reporting of actual intake is more common in men and under-reporting in women [23]. Black’s adjustment [24] of Goldberg et al. [25] [was used to investigate possible misreporting of energy intake. Estimated energy intake (EEI) was calculated from the FFQ responses, while sex-specific resting energy expenditure (REE) was calculated using the Mifflin–St. Jeor equations [26], derived from the Harris–Benedict equations [27]. Study participants were also asked if they had started a diet or changed their diet in the previous 5 weeks to assess possible discrepancies between BMI and EEI.

#### 2.4.2. Missing Data

Individuals who did not complete the dietary assessment and/or questions pertaining to odor perception were excluded from the analysis. For individual missing data, which could be assumed missing at random, cases were omitted on an analysis-by-analysis basis.

## 3. Results

### 3.1. Study Population

**Figure 1:**
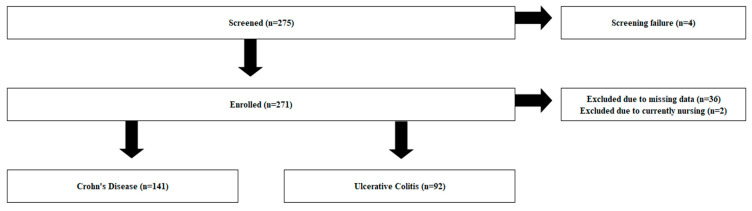
Flow chart of patient enrollment.

From 275 IBD individuals screened, n = 4 were defined as screening failures, resulting in n = 271 IBD patients enrolled in the study. Of those, n = 36 were excluded from this analysis due to missing data, while n = 2 were excluded due to nursing [Figure 1].

Sex distribution was well balanced (women n = 117 (50.2%)) with a skewed distribution of disease entities (Crohn’s disease n = 141 (60.5%)). Of the 233 cases analyzed, n = 117 (52.9%) were in remission, and the median age was 39 [IQR: 30–50]. A total of 53 individuals (22.7%) had been diagnosed with one or more food allergies or intolerances. Additionally, 103 individuals (44.2%) had previously undergone nutritional counselling due to their IBD (Table 1).

### 3.2. Healthy Controls

A total of 101 healthy individuals were screened. Of those n = 3 were excluded from further analysis due to missing data, and n = 2 were excluded due to nursing. Sex-distribution was skewed (women n = 67 (69.8%)), while median age was 30 [IQR: 23–39] (Table S3: Baseline characteristics of healthy controls).

### 3.3. Main Results

#### 3.3.1. IBD Cohort vs. Healthy Controls

**Figure 2:**
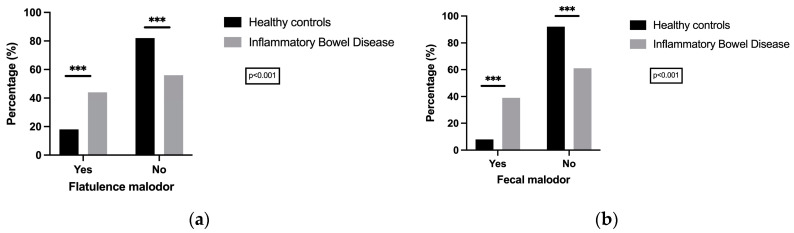
Percentual distribution of (**a**) flatulence malodor and (**b**) fecal malodor between IBD patients and healthy controls. Result of chi-square test shows significant differences in intestinal malodor perception between IBD patients and healthy controls (HCs) cohort for (**a**) flatulence malodor (*p* < 0.001) and (**b**) fecal malodor (*p* < 0.001). *** marks significance level *p* < 0.001.

As a preliminary investigation, the olfactory perception of fecal matter and flatulence has been compared between individuals diagnosed with IBD and a control group of healthy individuals. Notable differences in the distribution of malodorous flatulence and feces have been identified between the groups, with a clear tendency towards higher levels of malodorous flatulence and feces in the IBD collective (Figure 2a,b).

To gain further insight into the potential association between dietary behavior and malodor perception, *t*-tests were conducted on a selection of dietary variables. The results indicated statistically significant differences both within and between the distinct groups. Starting with the analysis of dietary behavior and flatulence malodor perception, the difference in total daily food and beverage intake (g/d) in IBD was statistically significant (*p* = 0.009; g = −0.4). The malodorous group demonstrated a higher consumption of food and beverages per day (2718 (g/d) vs. 2165 (g/d)). Furthermore, the EEI (kJ/d) was observed to be elevated in this group (8773 (kJ/d) vs. 7588 (kJ/d); *p* = 0.030; g = −0.3). In the HC group, however, only the daily intake of fruits and nuts (g/d) was significant (*p* = 0.023; g = 0.5) between the odor perception groups. Here, the malodorous flatulence group consumed less on average per day (159 (g/d) vs. 242 (g/d)). The outcome-specific *t*-test between the groups showed significant differences, especially between HC and IBD without malodorous flatulence. Most pronounced differences between the groups have been found for sQ-HPF (*p* = 0.001; g = −0.5), MDS (*p* = 0.002; g = 0.4), Vegetables (*p* ≤ 0.001; g = 1.0), Legumes (*p* ≤ 0.001; g = 0.7), Meat (*p* ≤ 0.001; g = −0.5) and Poultry (*p* = 0.002; g = −0.4) consumption. For the flatulence malodor outcome, daily legume intake was significant (*p* = 0.018; g = 0.9), as were daily HPF energy expenditure (*p* = 0.008; g = −0.4), daily UPFD energy expenditure (*p* = 0.036; g = −0.3), and daily EEI (*p* = 0.036; g = −0.3) (Table 2).

For fecal malodor perception, the differences in the sQ-HPF score were significant in both the IBD and HC cohorts (IBD *p* = 0.020; g = −0.3; HC *p* = 0.009; g = −0.7). The group with the outcome of malodorous feces exhibited a higher score and, consequently, a higher proportion of highly processed foods in their daily diet (IBD = 7; HC = 7). No other significant difference was reported in the IBD cohort, whereas in the HC cohort the difference in daily energy intake from HPFs was significant (*p* = 0.018; g = −0.9) between the groups. The group exhibiting fecal malodor perception demonstrated a daily HPF energy intake of 5407 (kJ/d), while the group without fecal malodor exhibited a daily HPF energy intake of 3530 (kJ/d). The outcome-specific *t*-test between the groups revealed significant differences, particularly between the HC and IBD without fecal malodor groups. Most pronounced differences between the groups have been found for sQ-HPF (*p* = 0.005; g = −0.4), HPF (*p* =< 0.001; g = −0.5), MDS (*p* = 0.008; g = 0.4), Vegetables (*p* =< 0.001; g = 0.8), Legumes (*p* =< 0.001; g = 0.7), Meat (*p* =< 0.001; g = −0.4), and Poultry (*p* = 0.004; g = −0.4) consumption. With regard to the outcome of fecal malodor, only the daily legume intake exhibited a statistically significant difference between IBDs and HCs (*p* = 0.049; g = 1.2) [Table 3].

#### 3.3.2. Inflammation and Odor: Feces and Flatulence in IBD Patients

**Figure 3:**
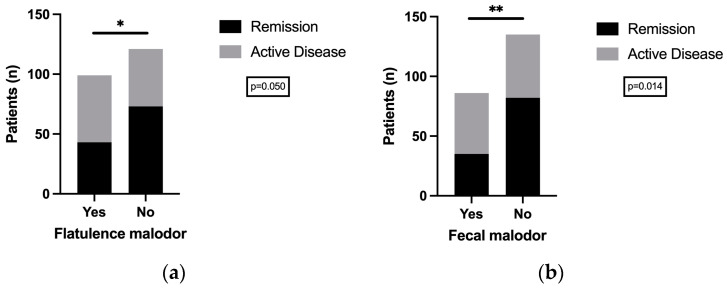
Distribution of IBD disease activity in comparison with (**a**) flatulence malodor and (**b**) fecal malodor. Result of Bonferroni correction to fisher’s exact test shows significant differences in intestinal malodor perception between IBD patients in remission vs. active disease for (**a**) flatulence malodor (*p* = 0.050) and (**b**) fecal malodor (*p* = 0.014). Total number of IBD patients is given as *n*. * marks significance level *p* < 0.05 and ** marks significance level *p* < 0.01.

To further evaluate the relationship between intestinal inflammation and fecal and flatulence malodor, additional analyses have been conducted. Calprotectin, an objective measure of intestinal inflammation, showed a significant difference between IBD patients with and without malodorous feces (*p* = 0.015; g = −0.4), but not between IBD patients with and without malodorous flatulence (*p* = 0.320; g = −0.1). (Figure S1a,b: Comparison of mean fecal calprotectin (mg/kg) between (a) flatulence malodor group and (b) fecal malodor group). Furthermore, the Bonferroni correction to fisher’s exact test showed statistical differences in the distribution of fecal and flatulence malodor between IBD patients in remission and those with an active disease status (fecal malodor: *p* = 0.014; flatulence malodor: *p* = 0.050) [Figure 3]. However, a subsequent analysis of the distribution of entity and remission status, stratified by malodor, revealed no statistically significant differences after the application of the Bonferroni correction. (Figure S2a–d: Distribution of entity and remission status, stratified by malodor) Therefore, logistic regression analysis was conducted to assess possible disease associations as well as dietary associations.

#### 3.3.3. Determining the Influence of Disease-Specific and Dietary-Specific Factors on Flatulence Odor Perception in Patients with IBD

An adjusted logistic regression analysis of the influence of disease-specific and dietary-specific factors on flatulence odor perception in patients with IBD showed no statistically significant risk factors apart from fecal odor perception (OR: 0.1; 95% CI: 0.03–0.11; *p* < 0.001; adjusted OR: 0.1; 95% CI: 0.03–0.11; *p* < 0.001) and the total daily amount of food and beverages (OR: 1.0; 95% CI: 1.00–1.00; *p* < 0.009; adjusted OR: 1.0; 95% CI: 1.00–1.00; *p* < 0.018) [Table 4].

#### 3.3.4. Determining the Influence of Disease-Specific and Dietary-Specific Factors on Fecal Odor Perception in Patients with IBD

An adjusted logistic regression analysis of the influence of disease-specific and dietary-specific factors on fecal odor perception in patients with IBD showed male sex as a statistically significant risk factors, the likelihood of fecal malodor was significantly increased in men (OR: 1.5; 95% CI: 0.89–2.56; *p* = 0.127; adjusted OR: 2.3; 95% CI: 1.13–4.61; *p* = 0.021). There were no further significant risk factors, apart from flatulence malodor (OR: 0.1; 95% CI: 0.03–0.11; *p* < 0.001; adjusted OR: 0.1; 95% CI: 0.02–0.11; *p* < 0.001) (Table 5).

## 4. Discussion

This comprehensive study aimed to investigate the relationship between dietary habits and perceived flatulence and fecal malodor in individuals with IBD and healthy controls. Furthermore, it sought to evaluate disease-specific parameters and potential associations with intestinal malodor in individuals with IBD. Percentual distribution of flatulence and fecal malodor perception between IBD patients and healthy controls showed vast differences, with further analysis revealing different dietary habits between the cohorts. It is well known that dietary behavior of IBD individuals differs from the general population [28,29], with IBD patients often modifying their diet habits after diagnosis, frequently avoiding certain foods [30] or adapting a low FODMAP diet [31]. This dietary adaptation may be indicated by the lower mean daily intake of legumes in the IBD cohort (16 g/d for no flatulence odor perception; 14 g/d for flatulence odor perception; *p* = 0.617; g = 0.1) compared with the healthy control group (41 g/d for no flatulence odor perception; 40 g/d for flatulence odor perception; *p* = 0.954; g = 0.0). While difficult-to-digest foods, such as legumes and cruciferous vegetables, are thought to contribute to increasing intestinal gas production [31], this difference in mean daily intake between IBD and HC cohorts for the flatulence malodor group was not only statistically but also clinically significant, as indicated by the effect size (*p* = 0.018; g = 0.9). In the analysis of fecal malodor between IBD and HC, this was also the case (*p* = 0.049; g = 1.2). As microbiome changes in IBD may contribute to this effect, different factors, most likely disease-specific, may influence malodors in IBD [32]. In individuals with IBD, the gut microbiome tends to exhibit reduced diversity and shows greater susceptibility to compositional shifts over time [33,34,35]. Moreover, changes in microbiota composition have been documented during both flare-ups and periods of clinical remission in inflammatory bowel disease [36,37]. These variations in microbial profiles may precede alterations in the biochemical disease trajectory and could potentially reflect underlying differences in fecal and flatulence malodor between IBD patients with different disease activity statuses, as well as between IBD patients and healthy controls. The observed variations in malodor between CD and UC individuals could potentially be accounted for by the distinction in the composition of the microbiome between these two entities, particularly during periods of remission [32,38]. Subsequent logistic regression analyses of fecal malodor in the IBD collective revealed a significant association with male sex. The perception of odor is subjective and therefore prone to bias. In addition, there are known sex differences in olfactory perception: women generally outperform men [39]. Individuals with IBD are accustomed to addressing inquiries pertaining to bowel movements, flatulence, and other physiological processes that are frequently regarded as taboo in public discourse. It seems implausible that women with IBD would not provide truthful responses. However, given that women in general tend to exhibit heightened levels of social desirability bias, it is plausible that reporting bias may be more prevalent among women in the healthy control group [23]. It is therefore not possible to distinguish with certainty between social and/or biological factors when investigating the association between male sex and the perception of fecal malodor in individuals with IBD.

The present study—to the best of our knowledge—represents the first comprehensive investigation into the relationship between dietary behavior, disease activity, and perceived intestinal malodor in subjects with IBD compared to healthy controls.

Our analysis is strengthened by several factors, including the comparison with a healthy control cohort, the comprehensive dietary analyses, the consideration of potential sources of bias, and the adjustment of the analysis for potential confounding factors. However, the analysis is also constrained by the skewed distribution of IBD entities, as well as the absence of objective measures of flatulence and fecal odor. To overcome these limitations, future studies on intestinal odor perception could make use of quantitative measures on flatulence and fecal characteristics as odor measuring devices, gas chromatography, or in vitro fermentation and gas capsule systems to measure and assess selected gas species. In addition, investigation of additional biological associations as potential links between dietary behavior and odor (e.g., microbial analysis) would be beneficial to gain a deeper understanding of the precise systemic relationships involved. As retrospective measures of dietary behavior, such as the FFQ, may introduce recall bias, we adjusted for over- and under-reporting. Future studies should further highlight these limitations and implement strategies to objectively measure and validate dietary habits (e.g., 24 h dietary recall, objective dietary measurement tools such as urine and blood biomarkers). Moreover, as this is a monocentric setting, certain recruitment biases inherent to a tertiary referral center cannot be discounted. Future studies should aim for multicentric settings to broaden the generalizability of these findings.

## Figures and Tables

**Table 1:** Baseline characteristics.

		Baseline
		(n = 233)
Demographics [Md[IQR] or n(%)]		
Disease entity	Crohn‘s disease	141 (60.5%)
	Ulcerative colitis	92 (39.5%)
Women		117 (50.2%)
Remission		117 (52.9%)
Location of Crohn’s	L1:	35 (24.8%)
	L2:	25 (17.7%)
	L3:	67 (47.5%)
	L4:	14 (9.9%)
Crohn’s behavior	B1:	51 (36.2%)
	B2:	66 (46.8%)
	B3:	24 (17%)
UC Montreal classification	Proctitis	6 (6.5%)
	Left–sided colitis	32 (34.8%)
	Pancolitis	54 (58.7%)
MUST	Low Risk	128 (54.9%)
	Medium Risk	49 (21%)
	High Risk	56 (24%)
One or more diagnosed food allergies/intolerances		53 (22.7%)
Disease duration in years		12.42 [6.84–19.5]
Nutritional counselling in the past due to IBD		103 (44.2%)
Gastrointestinal surgery		85 (36.5%)
BMI (kg/m^2^)		24.16 [21.45–27.77]
Age (years)		39 [30–50]
Smoking status (current or former)		81 (34.9%)
Calprotectin (mg/kg)		92 [27.85–498.5]
C–reactive protein (mg/l)		1.7 [0.75–4.8]

Baseline characteristics of study participant demographic data are reported as totals and proportions [n(%)], or median and interquartile range [Md(IQR)]. UC = ulcerative colitis; MUST = malnutrition universal screening tool; BMI = body mass index; L1 = ileal; L2 = colonic; L3 = ileocolonic; L4 = isolated upper disease; B1 = non-stricturing, non-penetrating; B2 = stricturing; B3 = penetrating.

**Table 2:** Associations between dietary behavior and flatulence malodor perception in individuals with IBD and healthy controls.

		FLATULENCE													
		IBD−Cohort						Healthy Controls							
Dietary Variables	Malodor	n	Mean	SD	SEM	p_t−test_	*g*	n	Mean	SD	SEM	p_t−test_	*g*	p_t−test: Cohort_	*g*
sQ−HPF	No	130	6	3	0	0.212	−0.2	76	5	3	0	0.056	−0.5	**0.001**	−0.5
	Yes	102	7	3	0			17	6	3	1			0.635	−0.1
MDS	No	129	4	2	0	0.991	0.0	73	5	2	0	0.223	0.3	**0.002**	0.4
	Yes	100	4	2	0			17	4	2	0			0.731	0.1
Food and Beverages (g/d)	No	130	2165	1410	124	**0.009**	−0.4	74	2333	1070	124	0.204	−0.5	0.374	0.1
	Yes	102	2718	1692	168			17	2966	1917	465			0.583	0.1
Vegetables (g/d)	No	130	111	125	11	0.986	0.0	74	273	215	25	0.286	0.3	**<0.001**	1.0
	Yes	102	111	114	11			17	212	193	47			0.050	0.8
Legumes (g/d)	No	130	16	29	3	0.617	0.1	74	41	42	5	0.954	0.0	**<0.001**	0.7
	Yes	102	14	27	3			17	40	39	10			**0.018**	0.9
Fruits and nuts (g/d)	No	130	158	157	14	0.182	−0.2	74	242	189	22	**0.023**	0.5	**0.002**	0.5
	Yes	102	197	265	26			17	159	112	27			0.311	−0.2
Cereals (g/d)	No	130	223	146	13	0.403	−0.1	74	240	169	20	0.242	0.3	0.461	0.1
	Yes	102	240	151	15			17	190	91	22			0.191	−0.3
Fish (g/d)	No	130	13	16	1	0.202	−0.2	74	11	20	2	0.365	−0.2	0.432	−0.1
	Yes	102	16	20	2			17	16	10	3			0.908	0.0
Meat (g/d)	No	130	66	68	6	0.201	−0.2	74	35	32	4	0.150	−0.7	**<0.001**	−0.5
	Yes	102	77	64	6			17	65	81	20			0.485	−0.2
Poultry (g/d)	No	130	31	33	3	0.486	−0.1	74	18	25	3	0.078	−0.5	**0.002**	−0.4
	Yes	102	37	84	8			17	31	38	9			0.773	−0.1
Dairy products (g/d)	No	130	240	246	22	0.115	−0.2	74	313	265	31	0.272	−0.3	**0.048**	0.3
	Yes	102	299	326	32			17	406	469	114			0.246	0.3
Ethanol (g/d)	No	130	46	134	12	0.865	0.0	74	88	217	25	0.627	0.1	0.136	0.2
	Yes	102	49	82	8			17	62	105	25			0.568	0.1
HPF (g/d)	No	130	821	954	84	0.112	−0.2	74	588	645	75	0.140	−0.4	0.063	−0.3
	Yes	102	1075	1358	134			17	867	888	215			0.544	−0.2
HPF (kJ/d)	No	117	4826	2951	273	0.248	−0.2	69	3668	2312	278	0.684	−0.1	**0.006**	−0.4
	Yes	96	5348	3623	370			16	3915	1461	365			**0.008**	−0.4
UPFD (g/d)	No	130	561	878	77	0.143	−0.2	74	395	585	68	0.099	−0.4	0.149	−0.2
	Yes	102	762	1143	113			17	689	910	221			0.804	−0.1
UPFD (kJ/d)	No	130	3049	2230	196	0.129	−0.2	74	2293	1666	194	0.346	−0.3	**0.007**	−0.4
	Yes	102	3591	3186	315			17	2697	1156	280			**0.036**	−0.3
EEI (kJ/d)	No	130	7588	3790	332	**0.030**	−0.3	74	7420	3077	358	0.907	0.0	0.746	0.0
	Yes	102	8773	4473	443			17	7328	2071	502			**0.036**	−0.3
REE (kJ/d)	No	130	6507	1081	95	0.598	−0.1	74	6308	1109	129	0.647	−0.1	0.212	−0.2
	Yes	102	6589	1265	125			17	6444	1076	261			0.623	−0.1

Results of Student’s *t*-test between malodor yes/no groups and the IBD and HC cohort are reported as arithmetic mean, standard deviation (SD), standard error of the mean (SEM), the level of significance (p), and the estimated effect size (g). P_t-test_ is printed bold when significant. Units of daily intake are reported as kilojoules (kJ) or grams (g) per day (d). IBD = inflammatory bowel disease; SD = standard deviation; SEM = standard error of the mean; sQ-HPF = screening questionnaire of highly processed food consumption; MDS = Mediterranean diet score. P_t-test_ is printed bold when significant.

**Table 3:** Associations between dietary behavior and fecal malodor perception in individuals with IBD and healthy controls.

		FECAL													
		IBD-Cohort						Healthy Controls							
Dietary Variables	Malodor	n	Mean	SD	SEM	p_t−test_	*g*	n	Mean	SD	SEM	p_t−test_	*g*	p_t−test: Cohort_	*g*
sQ−HPF	No	142	6	3	0	**0.020**	−0.3	87	5	3	0	**0.009**	−0.7	**0.005**	−0.4
	Yes	91	7	2	0			8	7	1	1			0.920	0.0
MDS	No	140	4	2	0	0.858	0.0	84	5	2	0	0.942	0.0	**0.008**	0.4
	Yes	90	4	2	0			8	5	2	1			0.290	0.4
Food and Beverages (g/d)	No	142	2264	1494	125	0.081	−0.2	85	2437	1261	137	0.856	−0.1	0.371	0.1
	Yes	91	2628	1638	172			8	2524	1558	551			0.862	−0.1
Vegetables (g/d)	No	142	120	141	12	0.270	0.1	85	263	210	23	0.440	0.3	**<0.001**	0.8
	Yes	91	102	95	10			8	203	221	78			0.238	0.9
Legumes (g/d)	No	142	16	32	3	0.737	0.0	85	40	42	5	0.937	0.0	**<0.001**	0.7
	Yes	91	15	19	2			8	39	29	10			**0.049**	1.2
Fruits and nuts (g/d)	No	142	176	193	16	0.937	0.0	85	231	184	20	0.267	0.4	**0.035**	0.3
	Yes	91	178	242	25			8	157	113	40			0.808	−0.1
Cereals (g/d)	No	142	229	149	12	0.904	0.0	85	223	145	16	0.082	−0.6	0.769	0.0
	Yes	91	231	149	16			8	323	239	84			0.114	0.6
Fish (g/d)	No	142	13	15	1	0.215	−0.2	85	10	12	1	0.273	−1.2	0.123	−0.2
	Yes	91	17	22	2			8	31	49	17			0.438	0.6
Meat (g/d)	No	142	66	68	6	0.173	−0.2	85	39	46	5	0.159	−0.5	**<0.001**	−0.4
	Yes	91	78	63	7			8	63	51	18			0.516	−0.2
Poultry (g/d)	No	142	30	29	2	0.297	−0.2	85	19	25	3	0.057	−0.7	**0.004**	−0.4
	Yes	91	40	90	9			8	38	47	17			0.954	0.0
Dairy products (g/d)	No	142	254	276	23	0.431	−0.1	85	331	321	35	0.657	0.2	0.056	0.3
	Yes	91	284	297	31			8	280	145	51			0.969	0.0
Ethanol (g/d)	No	142	51	134	11	0.579	0.1	85	66	171	19	0.186	−1.0	0.466	0.1
	Yes	91	42	73	8			8	254	361	128			0.141	1.8
HPF (g/d)	No	142	822	1076	90	0.075	−0.2	85	574	546	59	0.217	−1.1	**0.022**	−0.3
	Yes	91	1097	1248	131			8	1289	1485	525			0.682	0.2
HPF (kJ/d)	No	130	4838	3064	269	0.251	−0.2	79	3530	2007	226	**0.018**	−0.9	**<0.001**	−0.5
	Yes	84	5365	3573	390			8	5407	2855	1009			0.975	0.0
UPFD (g/d)	No	142	548	893	75	0.075	−0.3	85	395	523	57	0.298	−0.9	0.150	−0.2
	Yes	91	802	1145	120			8	964	1428	505			0.706	0.1
UPFD (kJ/d)	No	142	3090	2256	189	0.184	−0.2	85	2290	1565	170	0.204	−0.5	**0.002**	−0.4
	Yes	91	3572	3262	342			8	3031	1620	573			0.645	−0.2
EEI (kJ/d)	No	142	7709	3836	322	0.074	−0.2	85	7221	2804	304	0.075	−0.7	0.272	−0.1
	Yes	91	8702	4512	473			8	9126	3447	1219			0.796	0.1
REE (kJ/d)	No	142	6485	1188	100	0.371	−0.1	85	6303	1099	119	0.260	−0.4	0.250	−0.2
	Yes	91	6625	1122	118			8	6770	1261	446			0.731	0.1

Results of Student’s *t*-test between malodor yes/no groups and the IBD and HC cohort are reported as arithmetic mean, standard deviation (SD), standard error of the mean (SEM), the level of significance (p), and the estimated effect size (g). P_t-test_ is printed bold when significant. Units of daily intake are reported as kilojoules (kJ) or grams (g) per day (d). IBD = inflammatory bowel disease; SD = standard deviation; SEM = standard error of the mean; sQ-HPF = screening questionnaire of highly processed food consumption; MDS = Mediterranean diet score. P_t-test_ is printed bold when significant.

**Table 4:** Fully adjusted logistic regression analysis of the influence of disease-specific and dietary-specific factors on flatulence malodor in IBD patients.

		Outcome: Flatulence Malodor		
		n	Fully Adjusted OR [95%CI]	*p*
Entity	CD (1)	134	0.5 [0.23–0.99]	0.046
	UC	86		
Strong faecal odor perception	No	134	0.1 [0.03–0.11]	<0.001
	Yes (1)	86		
Total daily amount of food and beverages (g/d)		220	1.0 [1.00–1.00]	0.019
Vegetarian	No	194	0.3 [0.09–0.75]	0.012
	Yes (1)	26		

Results of logistic regression analysis (univariate and adjusted (multivariate)) are reported as the odds ratio (OR), 95% confidence interval (CI), and level of significance (p). CD = Crohn’s disease; UC = ulcerative colitis. Adjustment factors for the fully adjusted model: Sex, BMI, Fiber, Protein, Sugar, Total daily amount of food and beverages (g/d), Legumes, Cereals, Fish, Meat, Dairy Products, Eggs, sQ-HPF score, Remission status, Entity, vegetarian diet, age, fecal malodor, pouch/stoma. Reference groups are indicated as (1).

**Table 5:** Fully adjusted logistic regression analysis of the influence of disease-specific and dietary-specific factors on fecal malodor in IBD patients.

		Outcome: Fecal Malodor		
		n	Fully Adjusted OR [95%CI]	*p*
Sex	Men	111	2.1 [1.01–4.31]	0.047
	Women (1)	109		
Strong flatulence odor perception	No	121	0.0 [0.02–0.10]	<0.001
	Yes (1)	99		
sQ-HPF score		220	1.1 [1.00–1.31]	0.049

Results of logistic regression analysis (univariate and adjusted (multivariate)) are reported as the odds ratio (OR), 95% confidence interval (CI), and level of significance (p). Adjustment factors for the fully adjusted model: Sex, BMI, Fiber, Protein, Sugar, Total daily amount of food and beverages (g/d), Legumes, Cereals, Fish, Meat, Dairy Products, Eggs, sQ-HPF score, Remission status, Entity, vegetarian diet, age, flatulence malodor, pouch/stoma. Reference groups are indicated as (1).

## Data Availability

The original contributions presented in the study are included in the article, further inquiries can be directed to the corresponding author.

## Supplementary Materials

The following supporting information can be downloaded at: https://www.mdpi.com/article/10.3390/jcm14010137/s1, Table S1: Adjusted logistic regression analysis of the influence of disease-specific and dietary-specific factors on flatulence malodor in IBD patients; Table S2: Adjustment factors for Outcome: flatulence malodor; Table S3: Adjusted logistic regression analysis of the influence of disease-specific and dietary-specific factors on fecal malodor in IBD patients; Table S4: Adjustment factors for Outcome: fecal malodor; Table S5: Baseline characteristics of healthy controls. Figure S1a,b: Comparison of mean fecal calprotectin (mg/kg) levels between flatulence/fecal malodor group; Figure S2a–d: Distribution of entity and remission status, stratified by malodor.
