# Supplementary material for: Exploring Dietary- and Disease-Related Influences on Flatulence and Fecal Odor Perception in Inflammatory Bowel Disease
Source: J Clin Med. 2024 Dec 29;14(1):137. doi: 10.3390/jcm14010137 (PMC11720819; doi:10.3390/jcm14010137)
Supplement: Supplementary file 1 [file jcm-14-00137-s001.zip › jcm-3391577-supplementary.pdf]

Table S1: Adjusted logistic regression analysis of the influence of disease–specific and dietary–specific factors on flatulence malodor in IBD patients

Outcome: flatulence malodor

		**n**	**OR [95%CI]**	**p**	**Adjusted OR [95%CI]**	**p**
Entity	CD (1)<br>UC	140<br>92	0.5 [0.29 – 0.85]	**0.011**	0.5 [0.26 – 1.14]	0.104
ADT	No (1)<br>Yes	100<br>132	1.3 [0.78 – 2.25]	0.290	1.3 [0.60 – 2.64]	0.536
Gastrointestinale Surgery	No<br>Yes (1)	147<br>85	0.8 [0.44 – 1.30]	0.320	0.8 [0.35 – 2.01]	0.699
Remission	No<br>Yes (1)	104<br>116	2.0 [1.16 – 3.40]	**0.013**	1.7 [0.85 – 3.59]	0.130
Sex	Men<br>Women (1)	116<br>116	0.9 [0.52 – 1.46]	0.597	0.5 [0.26 – 1.05]	0.068
Strong faecal odor perception	No<br>Yes (1)	141<br>91	0.1 [0.03 – 0.11]	**<0.001**	0.1 [0.03 – 0.11]	**<0.001**
Calprotectin		208	1.0 [1.00 – 1.00]	0.322	1.0 [1.00 – 1.00]	0.269
Age		232	1.0 [0.97 – 1.00]	0.118	1.0 [0.96 – 1.00]	0.097
sQ–HPF		232	1.1 [0.97 – 1.17]	0.211	0.9 [0.82 – 1.07]	0.384
MDS		229	1.0 [0.85 – 1.17]	0.991	1.0 [0.82 – 1.28]	0.836
CRP		215	1.0 [0.99 – 1.03]	0.333	1.0 [0.97 – 1.04]	0.777
BMI		232	1.0 [0.97 – 1.06]	0.586	1.0 [0.97 – 1.10]	0.268
Legumes (g/d)		232	1.0 [0.99 – 1.01]	0.618	1.0 [0.98 – 1.01]	0.602
Meat (g/d)		232	1.0 [1.00 – 1.01]	0.207	1.0 [1.00 – 1.01]	0.577
Dairy (g/d)		232	1.0 [1.00 – 1.00]	0.123	1.0 [1.00 – 1.00]	0.125
Ethanol (g/d)		232	1.0 [1.00 – 1.00]	0.865	1.0 [1.00 – 1.00]	0.236
Fiber (g/d)		232	1.0 [1.00 – 1.05]	0.086	1.0 [0.99 – 1.06]	0.194
Protein (g/d)		232	1.0 [1.00 – 1.02]	**0.017**	1.0 [1.00 – 1.02]	0.171
Sugar (g/d)		232	1.0 [1.00 – 1.01]	0.067	1.0 [0.99 – 1.01]	0.747
Eggs (g/d)		232	1.0 [0.99 – 1.01]	0.618	1.0 [0.99 – 1.01]	0.891
Total daily amount of food and beverages (g/d)		232	1.0 [1.00 – 1.00]	**0.009**	1.0 [1.00 – 1.00]	**0.018**
Vegetarian	No<br>Yes (1)	204<br>28	0.6 [0.29 – 1.42]	0.277	0.4 [0.13 – 1.03]	0.056

Results of logistic regression analysis (univariate and adjusted (multivariate)) are reported as the odds ratio (OR), 95% confidence interval (CI), and level of significance (p). Level of significance (p) is printed bold when significant. CD = Crohn's disease; UC = ulcerative colitis; ADT = advanced therapies; sQ–HPF = screening questionnaire of highly processed food consumption; MDS = Mediterranean diet score; CRP = c–reactive protein; BMI = body mass index; OR = odds ratio; CI = confidence interval.

Table S2: Adjustment factors for Outcome: flatulence malodor

	Adjustment factors for Outcome: flatulence malodor
Entity	Sex, Age, BMI, stool odor, ADT, remission, entity, Pouch/stoma
ADT	Sex, Age, BMI, stool odor, ADT, remission, entity, Pouch/stoma
Gastrointestinale Surgery	Sex, Age, BMI, stool odor, ADT, remission, entity, GI surgery
Remission	Sex, Age, BMI, stool odor, ADT, remission, entity, Pouch/stoma
Sex	Sex, Age, BMI, stool odor, Pouch/stoma
Strong faecal odor perception	Sex, Age, BMI, stool odor, ADT, remission, entity, Pouch/stoma
Calprotectin	Sex, Age, BMI, stool odor, Pouch/stoma, remission, Calpro
Age	Sex, Age, BMI, stool odor, Pouch/stoma
sQ–HPF	Sex, Age, BMI, stool odor, Pouch/stoma, vegetarian, Total daily amount of food and beverages (g/d), sQ–HPF
MDS	Sex, Age, BMI, stool odor, Pouch/stoma, vegetarian, Total daily amount of food and beverages (g/d), MDS
CRP	Sex, Age, BMI, stool odor, Pouch/stoma,remission, CRP
BMI	Sex, Age, BMI, stool odor, Pouch/stoma, BMI
Legumes (g/d)	Sex, Age, BMI, stool odor, Pouch/stoma, vegetarian, Total daily amount of food and beverages (g/d), legumes
Meat (g/d)	Sex, Age, BMI, stool odor, Pouch/stoma, vegetarian, Total daily amount of food and beverages (g/d), meat
Dairy (g/d)	Sex, Age, BMI, stool odor, Pouch/stoma, vegetarian, Total daily amount of food and beverages (g/d), dairy
Ethanol (g/d)	Sex, Age, BMI, stool odor, Pouch/stoma, vegetarian, Total daily amount of food and beverages (g/d), ethanol
Fiber (g/d)	Sex, Age, BMI, stool odor, Pouch/stoma, vegetarian, Total daily amount of food and beverages (g/d), fiber
Protein (g/d)	Sex, Age, BMI, stool odor, Pouch/stoma, vegetarian, Total daily amount of food and beverages (g/d), protein
Sugar (g/d)	Sex, Age, BMI, stool odor, Pouch/stoma, vegetarian, Total daily amount of food and beverages (g/d), sugar
Eggs (g/d)	Sex, Age, BMI, stool odor, Pouch/stoma, vegetarian, Total daily amount of food and beverages (g/d), eggs
Total daily amount of food and beverages (g/d)	Sex, Age, BMI, stool odor, Pouch/stoma, vegetarian, Total daily amount of food and beverages (g/d)
Vegetarian	Sex, Age, BMI, stool odor, Pouch/stoma, vegetarian, Total daily amount of food and beverages (g/d)

Adjustment factors for the adjusted (multivariate) logistic regression analysis for the outcome: flatulence malodor. ADT = advanced therapies; sQ–HPF = screening questionnaire of highly processed food consumption; MDS = Mediterranean diet score; CRP = c–reactive protein; BMI = body mass index.

Table S3: Adjusted logistic regression analysis of the influence of disease–specific and dietary–specific factors on fecal malodor in IBD patients

Outcome: Fecal malodor

		n	OR [95%CI]	p	Adjusted OR [95%CI]	p
Entity	CD (1)	141	0.6 [0.34 – 1.02]	0.058	0.7 [0.31 – 1.43]	0.299
	UC	92				
ADT	No (1)	101	1.1 [0.65 – 1.89]	0.695	1.1 [0.51 – 2.26]	0.851
	Yes	132				
Gastrointestinale Surgery	No	148	0.6 [0.34 – 1.02]	0.059	0.8 [0.34 – 1.88]	0.606

	Yes (1)	85				
Remission	No	104	2.3 [1.30 – 3.91]	**0.004**	2.0 [0.98 – 4.26]	0.057
	Yes (1)	117				
Sex	Men	116	1.5 [0.89 – 2.56]	0.127	2.3 [1.13 – 4.61]	**0.021**
	Women (1)	117				
Strong flatulence odor perception	No	130	0.1 [0.03 – 0.11]	**<0.001**	0.1 [0.02 – 0.11]	**<0.001**
	Yes (1)	102				
Calprotectin		208	1.0 [1.00 – 1.00]	**0.011**	1.0 [1.00 – 1.00]	**0.017**
Age		233	1.0 [0.98 – 1.01]	0.457	1.0 [0.98 – 1.03]	0.659
sQ–HPF		233	1.1 [1.02 – 1.24]	**0.022**	1.1 [0.98 – 1.30]	0.089
MDS		230	1.0 [0.86 – 1.19]	0.857	1.0 [0.82 – 1.28]	0.814
CRP		216	1.0 [0.99 – 1.03]	0.326	1.0 [0.98 – 1.03]	0.792
BMI		233	1.0 [0.95 – 1.05]	0.867	1.0 [0.92 – 1.04]	0.462
Legumes (g/d)		233	1.0 [0.99 – 1.01]	0.736	1.0 [0.99 – 1.01]	0.992
Meat (g/d)		233	1.0 [1.00 – 1.01]	0.179	1.0 [0.99 – 1.01]	0.762
Dairy (g/d)		233	1.0 [1.00 – 1.00]	0.431	1.0 [1.00 – 1.00]	0.480
Ethanol (g/d)		233	1.0 [1.00 – 1.00]	0.583	1.0 [0.99 – 1.00]	0.071
Fiber (g/d)		233	1.0 [0.98 – 1.03]	0.578	1.0 [0.96 – 1.03]	0.615
Protein (g/d)		233	1.0 [1.00 – 1.02]	**0.032**	1.00 [0.99 – 1.01]	0.970
Sugar (g/d)		233	1.0 [1.00 – 1.01]	0.087	1.0 [1.00 – 1.01]	0.681
Eggs (g/d)		233	1.0 [0.99 – 1.01]	0.530	1.0 [0.99 – 1.01]	0.927
Total daily amount of food and beverages (g/d)		233	1.0 [1.00 – 1.00]	0.085	1.0 [1.00 – 1.00]	0.891
Vegetarian	No	205	1.4 [0.61 – 3.26]	0.426	2.4 [0.84 – 6.71]	0.103
	Yes	28				

Results of logistic regression analysis (univariate and adjusted (multivariate)) are reported as the odds ratio (OR), 95% confidence interval (CI), and level of significance (p). Level of significance (p) is printed bold when significant. CD = Crohn's disease; UC = ulcerative colitis; ADT = advanced therapies; sQ–HPF = screening questionnaire of highly processed food consumption; MDS = Mediterranean diet score; CRP = c–reactive protein; BMI = body mass index; OR = odds ratio; CI = confidence interval.

Table S4: Adjustment factors for Outcome: fecal malodor

	Adjustment factors for Outcome: fecal malodor
Entity	Sex, Age, BMI, gas odor, ADT, remission, entity, Pouch/stoma
ADT	Sex, Age, BMI, gas odor, ADT, remission, entity, Pouch/stoma
Gastrointestinale Surgery	Sex, Age, BMI, gas odor, ADT, remission, entity, GI surgery
Remission	Sex, Age, BMI, gas odor, ADT, remission, entity, Pouch/stoma
Sex	Sex, Age, BMI, gas odor, Pouch/stoma
Strong flatulence odor perception	Sex, Age, BMI, gas odor, ADT, remission, entity, Pouch/stoma
Calprotectin	Sex, Age, BMI, gas odor, Pouch/stoma, remission, Calpro
Age	Sex, Age, BMI, gas odor, Pouch/stoma
sQ–HPF	Sex, Age, BMI, gas odor, Pouch/stoma, vegetarian, Total daily amount of food and beverages (g/d), sQ–HPF

MDS	Sex, Age, BMI, gas odor, Pouch/stoma, vegetarian, Total daily amount of food and beverages (g/d), MDS
CRP	Sex, Age, BMI, gas odor, Pouch/stoma, remission, CRP
BMI	Sex, Age, BMI, gas odor, Pouch/stoma
Legumes (g/d)	Sex, Age, BMI, gas odor, Pouch/stoma, vegetarian, Total daily amount of food and beverages (g/d), legumes
Meat (g/d)	Sex, Age, BMI, gas odor, Pouch/stoma, vegetarian, Total daily amount of food and beverages (g/d), meat
Dairy (g/d)	Sex, Age, BMI, gas odor, Pouch/stoma, vegetarian, Total daily amount of food and beverages (g/d), dairy
Ethanol (g/d)	Sex, Age, BMI, gas odor, Pouch/stoma, vegetarian, Total daily amount of food and beverages (g/d), ethanol
Fiber (g/d)	Sex, Age, BMI, gas odor, Pouch/stoma, vegetarian, Total daily amount of food and beverages (g/d), fiber
Protein (g/d)	Sex, Age, BMI, gas odor, Pouch/stoma, vegetarian, Total daily amount of food and beverages (g/d), protein
Sugar (g/d)	Sex, Age, BMI, gas odor, Pouch/stoma, vegetarian, Total daily amount of food and beverages (g/d), sugar
Eggs (g/d)	Sex, Age, BMI, gas odor, Pouch/stoma, vegetarian, Total daily amount of food and beverages (g/d), eggs
Total daily amount of food and beverages (g/d)	Sex, Age, BMI, gas odor, Pouch/stoma, vegetarian, Total daily amount of food and beverages (g/d)
Vegetarian	Sex, Age, BMI, gas odor, Pouch/stoma, vegetarian, Total daily amount of food and beverages (g/d)

Adjustment factors for the adjusted (multivariate) logistic regression analysis for the outcome: fecal malodor. ADT = advanced therapies; sQ–HPF = screening questionnaire of highly processed food consumption; MDS = Mediterranean diet score; CRP = c–reactive protein; BMI = body mass index.

Table S5: Baseline characteristics of healthy controls

Demographics [Md[IQR] or n(%)]		**Baseline (n=96)**
Women		67 (69.8%)
MUST	Low Risk	42 (43.8%)
	Medium Risk	33 (34.4%)
	High Risk	21 (21.9%)
One or more diagnosed food allergies/intolerances		11 (11.5%)
Smoking status (current or former)		20 (20.8%)
Age (years)		30 [23 – 39]
BMI (kg/m^2)		22.89 [20.98 – 26.08]

Baseline characteristics of healthy controls demographic data are reported as totals and proportions [n(%)], or median and interquartile range [Md(IQR)]. MUST = malnutrition universal screening tool; BMI = body mass index.

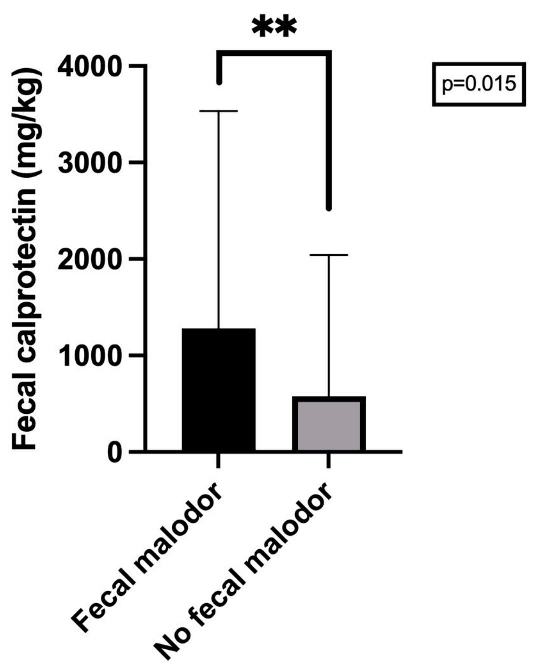

Figure S1a: Comparison of mean fecal calprotectin (mg/kg)levels between fecal malodor group.

Welch's t–test is significant (p=0.015).

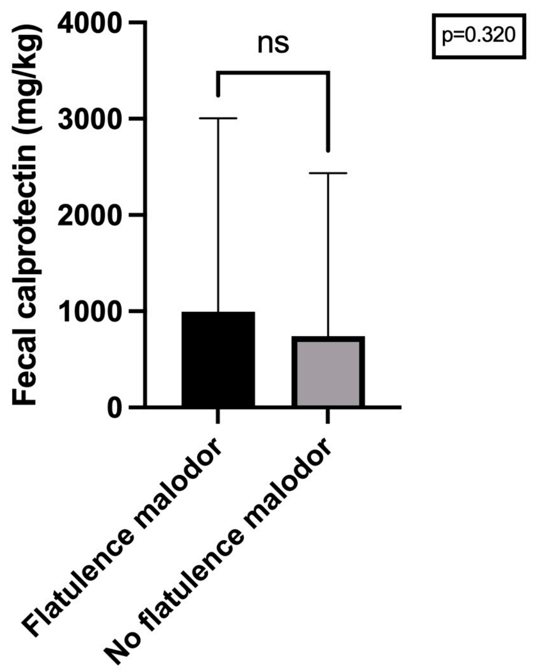

Figure S1b: Comparison of mean flatulence calprotectin (mg/kg) levels between flatulence malodor group.

Welch's t–test is not significant (p=0.320).

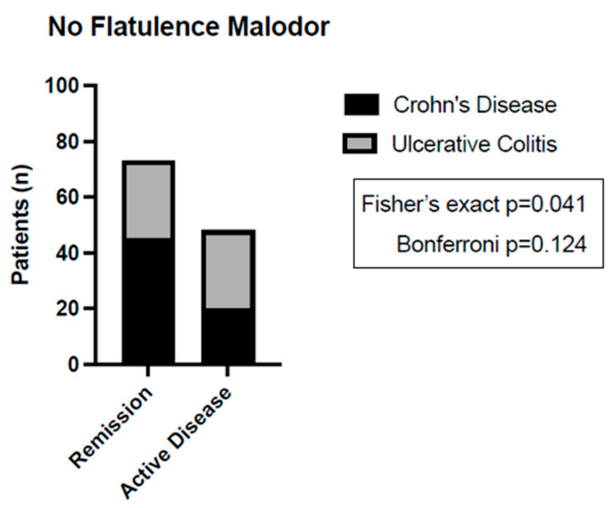

Figure S2a: Distribution of entity and remission status, stratified by malodor.

Fisher's exact test for the no flatulence malodor group is significant (p=0.041), however subsequent Bonferroni correction is not (p=0.124).

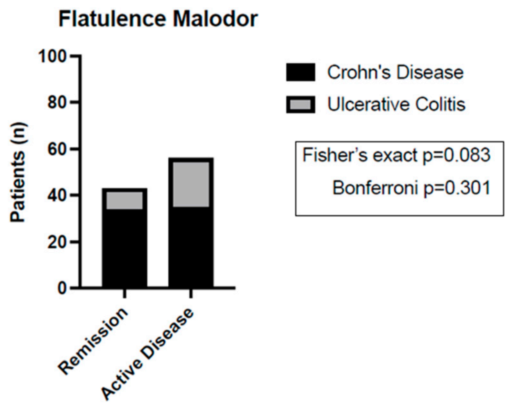

Figure S2b: Distribution of entity and remission status, stratified by malodor.

Fisher's exact test for the flatulence malodor group is not significant (p=0.083), neither is subsequent Bonferroni correction (p=0.301).

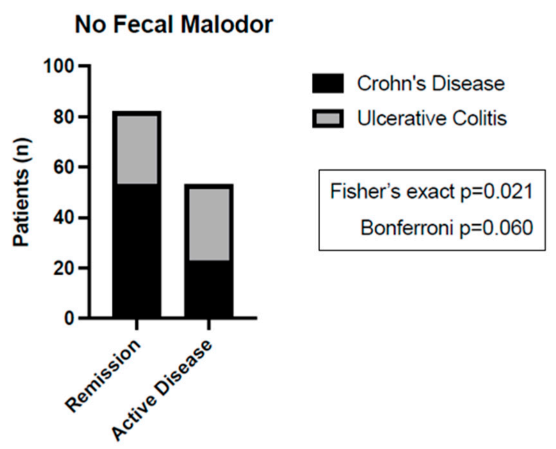

Figure S2c: Distribution of entity and remission status, stratified by malodor.

Fisher's exact test for the no fecal malodor group is significant (p=0.021), however subsequent Bonferroni correction is not (p=0.060).

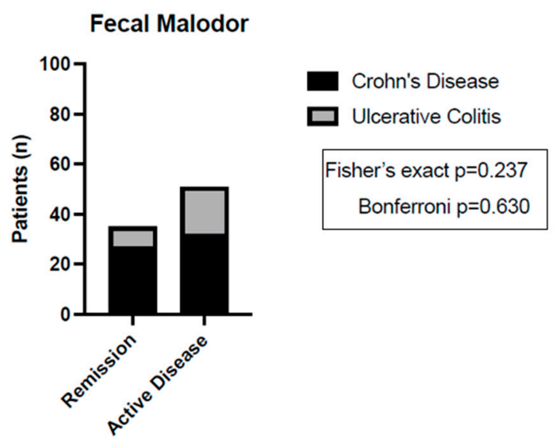

Figure S2d: Distribution of entity and remission status, stratified by malodor.

Fisher's exact test for the fecal malodor group is not significant (p=0.237), neither is subsequent Bonferroni correction (p=0.630).
